# Central Role of SREBP-2 in the Pathogenesis of Osteoarthritis

**DOI:** 10.1371/journal.pone.0035753

**Published:** 2012-05-25

**Authors:** Fotini Kostopoulou, Vasiliki Gkretsi, Konstantinos N. Malizos, Dimitrios Iliopoulos, Pagona Oikonomou, Lazaros Poultsides, Aspasia Tsezou

**Affiliations:** 1 Department of Cytogenetics and Molecular Genetics, University of Thessaly School of Medicine, Larissa, Greece; 2 Institute of Biomedical Research and Technology, Center for Research and Technology-Thessaly, Larissa, Greece; 3 Department of Orthopaedics, University of Thessaly School of Medicine, Larissa, Greece; 4 Department of Cancer Immunology & AIDS, Dana-Farber Cancer Institute and Department of Pathology, Harvard Medical School, Boston, Massachusetts, United States of America; 5 Department of Biology, University of Thessaly School of Medicine, Larissa, Greece; University of Western Ontario, Canada

## Abstract

**Background:**

Recent studies have implied that osteoarthritis (OA) is a metabolic disease linked to deregulation of genes involved in lipid metabolism and cholesterol efflux. Sterol Regulatory Element Binding Proteins (SREBPs) are transcription factors regulating lipid metabolism with so far no association with OA. Our aim was to test the hypothesis that SREBP-2, a gene that plays a key role in cholesterol homeostasis, is crucially involved in OA pathogenesis and to identify possible mechanisms of action.

**Methodology/Principal Findings:**

We performed a genetic association analysis using a cohort of 1,410 Greek OA patients and healthy controls and found significant association between single nucleotide polymorphism (SNP) 1784G>C in SREBP-2 gene and OA development. Moreover, the above SNP was functionally active, as normal chondrocytes’ transfection with SREBP-2-G/C plasmid resulted in interleukin-1β and metalloproteinase-13 (MMP-13) upregulation. We also evaluated SREBP-2, its target gene 3-hydroxy-3-methylglutaryl-coenzymeA reductase (HMGCR), phospho-phosphoinositide3-kinase (PI3K), phospho-Akt, integrin-alphaV (ITGAV) and transforming growth factor-β (TGF-β) mRNA and protein expression levels in osteoarthritic and normal chondrocytes and found that they were all significantly elevated in OA chondrocytes. To test whether TGF-β alone can induce SREBP-2, we treated normal chondrocytes with TGF-β and found significant upregulation of SREBP-2, HMGCR, phospho-PI3K and MMP-13. We also showed that TGF-β activated aggrecan (ACAN) in chondrocytes only through Smad3, which interacts with SREBP-2. Finally, we examined the effect of an integrin inhibitor, cyclo-RGDFV peptide, on osteoarthritic chondrocytes, and found that it resulted in significant upregulation of ACAN and downregulation of SREBP-2, HMGCR, phospho-PI3K and MMP-13 expression levels.

**Conclusions/Significance:**

We demonstrated, for the first time, the association of SREBP-2 with OA pathogenesis and provided evidence on the molecular mechanism involved. We suggest that TGF-β induces SREBP-2 pathway activation through ITGAV and PI3K playing a key role in OA and that integrin blockage may be a potential molecular target for OA treatment.

## Introduction

Osteoarthritis (OA) is a complex degenerative joint disease with multifactorial aetiology. Several factors including genetic susceptibility, increased mechanical load, injuries and inflammation of the joint, as well as obesity have been long considered as important risk factors of the disease [1] leading to progressive cartilage loss, formation of osteophytes and other significant alterations in ligaments, menisci and adjacent muscles [2]. Interestingly, however, recent studies point to the direction that OA is rather a metabolic disease [3], [4], which has also been linked to deregulation of lipid metabolism genes. This aspect is strengthened by proteomic analysis studies which have revealed that numerous lipid metabolism-related proteins are differentially expressed in osteoarthritic cartilage compared to normal [5], [6]. In addition, recent work from our group has shown that oxidized low-density lipoprotein (Ox-LDL) is present in the synovial fluid and that its receptor, lectin-like oxidized low-density lipoprotein receptor 1 (LOX-1) is detected in cartilage from both weight-bearing and non-weight-bearing areas, whereas no LOX-1 expression was found in normal cartilage [7]. The presence of LOX-1 in chondrocytes indicates that chondrocytes are indeed capable of internalizing lipids. We have also recently shown that osteoarthritic chondrocytes present intracellular lipid accumulation and exhibit reduced expression of genes regulating reverse cholesterol transport, such as Apolipoprotein A1 (ApoA1), or liver X receptors (LXR α and LXR β) compared to normal chondrocytes [8].

Sterol Regulatory Element Binding Proteins (SREBPs) are transcription factors that bind to the sterol regulatory element DNA sequence and regulate lipid metabolism [8]. To date, three members of the SREBP family have been identified: SREBP-1a, SREBP-1c and SREBP-2 [9], [10], [11], [12]. Both SREBP-1a and 1c are isoforms encoded by the *srebp1* gene, whereas *srebp2* gene encodes only one isoform [13], [14]. SREBP-1c regulates genes of fatty acid and triglyceride metabolism, while SREBP-2 preferentially activates genes of cholesterol metabolism and biosynthesis, such as 3-hydroxy-3-methylglutaryl coenzyme A (HMG-CoA) reductase (HMGCR) [8] and SREBP-1a regulates both sets of genes. SREBPs are synthesized as inactive precursor proteins anchored to the membranes of the endoplasmic reticulum (ER) where they remain in the presence of cholesterol [15]. When the cell is in need of lipids, they are activated by a two-step proteolytic cleavage of the transcriptionally active NH_2_-terminal portion [16]. The COOH-terminal domain forms a tight complex with SREBP cleavage-activating protein (SCAP) which functions as the sterol sensor in this system [17].

It has been shown, that SNPs in SREBP genes are associated with diseases related to the metabolic syndrome [18], [19], [20], [21]. More specifically, SNP 1784G >C in SREBP-2 gene, which play a key role in cholesterol homeostasis, results in substitution of a glucine by an alanine at amino acid 595 of the SREBP-2 protein (G595A) and has been associated with intima-media thickness (IMT), a marker of atherosclerosis, total cholesterol levels in hypercholesterolaemic subjects and elevated plasma lipids levels [22], [23] .

Recent evidence suggests that SREBPs are activated by phosphoinositide 3-kinase (PI3K) and Akt, both of which are considered to be mainly implicated in cell survival signalling pathways with many implications in most multifactorial diseases such as cancer and diabetes [24]. PI3K is activated by growth factors, components of the extracellular matrix (ECM) and integrins; the heterodimeric transmembrane receptors linking ECM to the intracellular compartment of the cell. PI3K activation may lead to enhanced cell survival signalling (through activation of Akt and NFkB), enhanced proliferation and invasion (through inhibition of GSK3β) or induction of differentiation (through MAPK activation) depending on the downstream signals that get activated in each case [25]. Thus, judging by the complexity of the PI3K signalling pathway, it is not surprising that PI3K activation may confer different outcomes as far as cellular fate is concerned depending on the downstream signals that are being transmitted every time.

With regard to OA several studies have demonstrated an important role played by different integrins, the transmembrane receptors that interact with the extracellular matrix (ECM) and mediate various intracellular signalling [26], [27]. More specifically, it was recently shown in a proteomic study that integrin alpha V (ITGAV) is upregulated in osteoarthritic chondrocytes [5]. Interestingly, transforming growth factor beta (TGF-β), one of the integrin-induced growth factors was shown to be involved in bone tissue formation and differentiation, while its expression in subchondral bone and osteophytes was increased in OA patients compared to non-OA [28]. TGF-β signaling is mainly mediated by Smad proteins. More specifically, upon TGF-β binding to its receptor, Smads become phosphorylated and translocate into the nucleus where they regulate the expression of target genes [29]. Interestingly, aggrecan (ACAN), a proteoglycan that together with Type-II collagen (COL2A1) forms a major structural component of articular cartilage is known to be a direct target gene of Smad3 which was shown to interact with SREBP-2 [30].

In the present study we tested the hypothesis that SREBP-2 is crucially involved in OA pathogenesis by carrying out a genetic association study. Moreover, we have also identified the molecular mechanism by which SREBP-2 becomes activated in osteoarthritic chondrocytes.

## Results

### Association Analysis: *1784G>C* Single Nucleotide Polymorphism (SNP) in SREBP-2 gene is Associated with OA Development

Since SREBPs seem to play a central role in regulating intracellular lipid metabolism, single nucleotide polymorphisms (SNPs) in these genes may interfere with lipid metabolism and associated disease conditions. Thus, in the present study, we investigated the association between SNP *1784G>*C (rs2228314) in SREBP-2 gene and OA development. We found a significant difference in the distribution of GC genotype (p<0.001; OR = 1.481) (Table 1) as well as in the allelic frequencies (p = 0.005; OR = 1.275) (Table 2) between patient and control group. In addition a significant correlation was observed between GC genotype and G, C alleles and Body Mass Index (BMI) (p<0.001; OR = 1.311) as well as K/L score (p = 0.026; OR = 1.289), whereas when sex stratification was performed, no significant difference was observed in the distribution of genotypic and allelic frequencies. Adjustment for risk factors, as BMI, age, and sex showed that significance was retained for BMI (p<0.05, OR = 1.25) and K/L score (p<0.05, OR = 1.243). All above indicate that *1784G>*C SNP is associated with increased BMI in OA and with OA severity by K/L score.

**Table 1:** Genotypes of 1784G>C (rs2228314) polymorphism in SREBP-2 gene.

Reference SNP ID	Genotype	Frequency no. (%)		p value	OR (95% CI)
		Patients (n = 709)	Controls (n = 701)		
rs2228314	GG	320 (45.1%)	385 (55%)		
	GC	384 (54.1%)	307 (43.8%)	p<0.001	1.481 (1.201–1.827)
	CC	5 (0.8%)	9 (1.2%)	p = 0.187	2.038 (0.693–5.992)

**Table 2:** Alleles of 1784G>C (rs2228314) polymorphism in SREBP-2 gene.

Reference SNP ID	Allele	Frequency no. (%)		p value	OR (95% CI)
		Patients (n = 1418)	Controls (n = 1402)		
rs2228314	G	1024 (72%)	1077 (77%)		
	C	394 (28%)	325 (23%)	p = 0.005	1.275 (1.076–1.511)

#### Power of the study

To insure that we had adequate power we calculated the minimum detectable ORs that could lead to a 95% probability of rejection of the full hypothesis of no association at a significance level of 0.05. The minimum detectable OR under the log additive model with power 95% and a significance level of 5% was calculated using Quanto version 1.2.4 and was found to be 1.54 for SREBP-2 (1784 G>C).

### Type II Collagen and Type I Collagen Ratio in OA and Normal Chondrocytes

**Figure 1:**
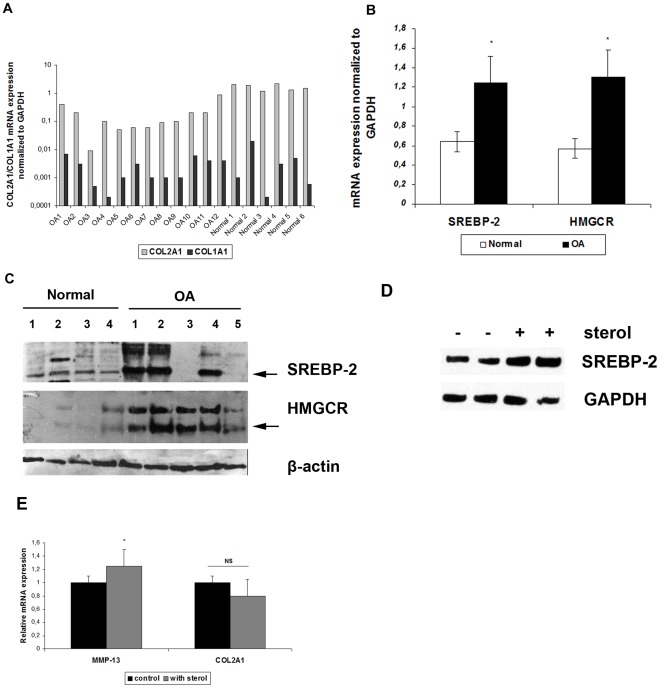
SREBP-2 and HMGCR mRNA and protein expression in normal and osteoarthritic chondrocytes. (**A**) COL2A1 and COL1A1 ratio was screened for the verification of chondrocytes’ phenotype in cultures (**B**) SREBP-2 and HMGCR mRNA expression in normal and osteoarthritic chondrocytes. GAPDH was used for normalization of the Real Time PCR data. All experiments were performed in duplicate. *p<0.05 (**C**) Representative film showing SREBP-2 and HMGCR protein expression evaluated by Western blot analysis in normal and osteoarthritic chondrocytes. β-actin was used as loading control. (**D**) Representative film showing SREBP-2 protein expression evaluated by Western blot analysis in osteoarthritic chondrocytes treated with 25 µΜ of 25-hydroxycholesterol. GAPDH was used as loading control. (**E**) MMP-13 and COL2A1 mRNA expression in osteoarthritic chondrocytes treated with 25 µΜ of 25-hydroxycholesterol. GAPDH was used for normalization of the Real Time PCR data. All experiments were performed in duplicate. *p<0.05, NS: non significant.

All OA and normal cartilage samples had significantly higher COL2A1 mRNA expression levels compared to COL1A1, verifying the chondrocytes’ phenotype in the cultures (Figure 1A).

### SREBP-2 and 3-hydroxy-3methylglutaryl Coenzyme A Reductase (HMGCR) mRNA and Protein Expression Levels are Elevated in OA Chondrocytes

Since SNP 1784G>C in SREBP-2 was found to be associated with OA we were tempted to investigate whether SREBP-2 mRNA and protein expression is altered in OA chondrocytes compared to normal. We evaluated SREBP-2 and its’ target gene, HMGCR mRNA and protein expression levels in osteoarthritic and normal chondrocytes and found that they were both significantly elevated in OA chondrocytes compared to normal (p<0.05) (Figures 1B and 1C).

### 25-hydroxycholesterol Treatment in OA Chondrocytes Resulted in SREBP-2 Upregulation

We treated OA chondrocytes with 25 µM 25-hydroxycholesterol for l2h in order to evaluate its effect on SREBP-2 expression and we found an increase in SREBP-2 and MMP-13 expression (Figures 1D and 1E).

### Phospho-PI3K and Phospho-Akt Protein Expression Levels are Elevated in OA Chondrocytes

**Figure 2:**
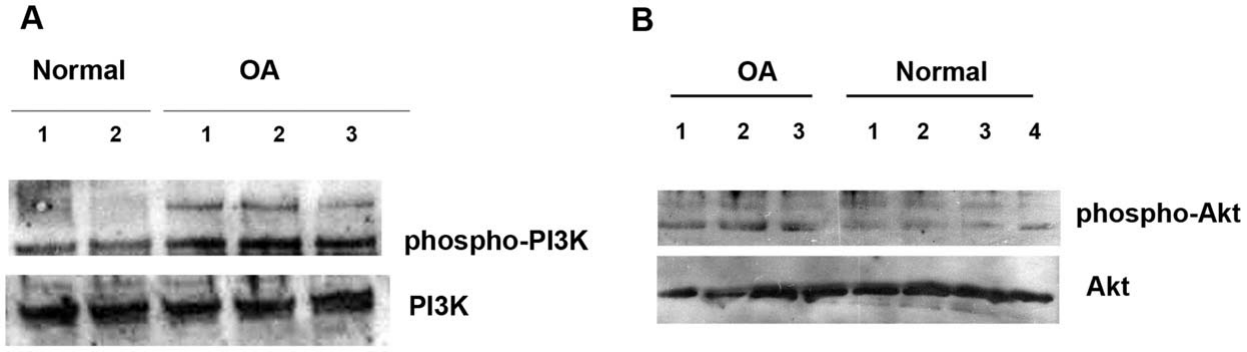
Phospho-PI3K and phospho-Akt expression in normal and osteoarthritic chondrocytes. (**A**) A representative western blot showing phospho-PI3K protein expression level in normal and osteoarthritic chondrocytes. Total PI3K expression level is shown in the lower panel. (**B**) A representative western blot showing phospho-Akt protein expression level in normal and osteoarthritic chondrocytes. Total Akt expression level is shown in the lower panel.

As recent evidence [24] suggests that PI3K and Akt activate SREBP-2, we studied the expression levels of phospho-PI3K and phospho-Akt in osteoarthritic and normal chondrocytes. We found that osteoarthritic chondrocytes had significantly higher phospho-PI3K and phospho-Akt protein expression compared to normal (p<0.05), while total PI3K and Akt expression levels remained stable (Figures 2A and 2B).

### TGF-β is Overexpressed in OA Chondrocytes while it also Induces SREBP-2 and HMGCR Upregulation in Normal Chondrocytes

**Figure 3:**
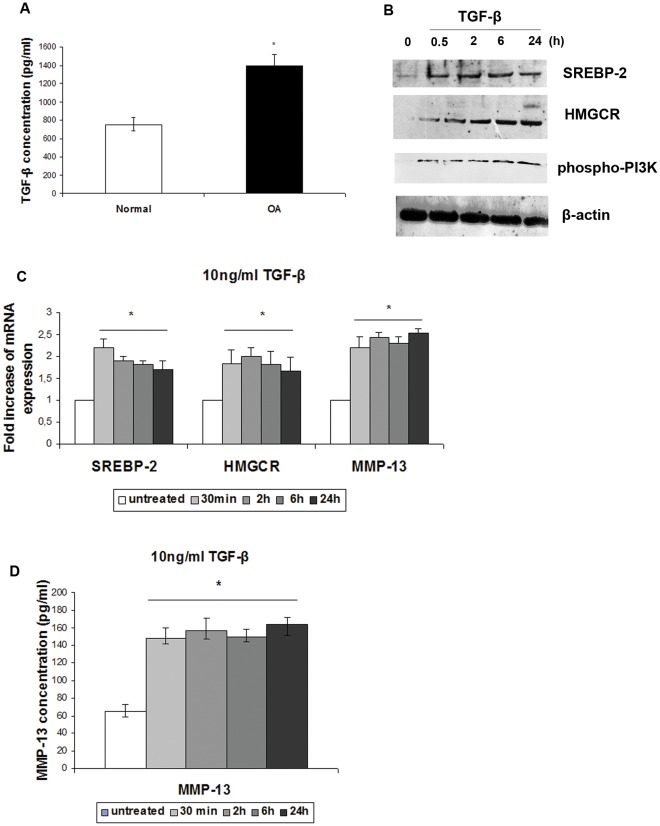
TGF-β expression in normal and osteoarthritic chondrocytes and effect of TGF-β treatment on SREBP-2, HMGCR, phospho-PI3K and MMP-13 expression. (**A**) TGF-β expression levels in normal and osteoarthritic chondrocytes detected by ELISA. *p<0.05 (**B**) Western blot showing SREBP-2, HMGCR and phospho-PI3K protein expression levels in normal and osteoarthritic chondrocytes treated with 10 ng/ml TGF-β for 0.5, 2, 6, and 24 h. β-actin was used as loading control. The same membrane was reprobed without stripping. (**C**) SREBP-2, HMGCR and MMP-13 mRNA in cultured normal chondrocytes following treatment with 10 ng/ml TGF-β for 0.5, 2, 6 and 24 h. All experiments were performed in duplicate and data are expressed as mean. *p<0.05 (**D**) MMP-13 protein expression levels in normal chondrocytes following treatment with 10 ng/ml TGF-β for 0.5, 2, 6 and 24 h detected by ELISA. *p<0.05.

PI3K, a protein playing a central role in multiple pathological and physiological conditions, is activated mainly by growth factors such as Transforming Growth Factor β (TGF-β), components of the ECM and their transmembrane receptors, integrins. To test how PI3K is activated in OA, we first performed ELISA assays to assess TGF-β expression level in OA and normal chondrocytes and found that TGF-β expression was elevated in osteoarthritic chondrocytes compared to normal (p<0.05) (Figure 3A). In order to test whether TGF-β can induce SREBP-2 activation by itself, normal chondrocytes were treated with 10 ng/ml TGF-β over different periods of time (from 0.5 to 24 h). Our results showed that both SREBP-2 and HMGCR mRNA and protein expression levels were upregulated in chondrocytes treated with TGF-β as opposed to untreated cells (p<0.05) (Figures 3B and 3C) indicating that there is a cause and effect relationship between TGF-β and SREBP-2/HMGCR. Moreover, we showed that TGF-β treatment upregulated phospho-PI3K (Figure 3B), and MMP-13 (Figure 3C and 3D) expression in normal chondrocytes, promoting thus the osteoarthritic phenotype.

### Pharmacological Inhibition of TGF-β Receptor Downregulates SREBP-2 Protein Expression but does not Lead to Restoration of their Normal Phenotype

**Figure 4:**
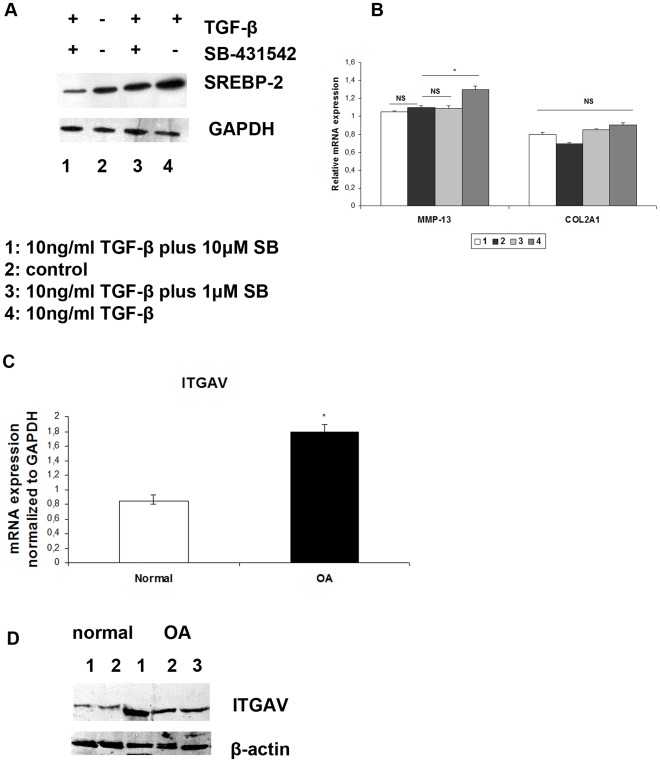
Effect of TGF-β receptor inhibitor (SB-431542) on SREBP-2 expression and ITGAV expression in normal and osteoarthritic chondrocytes. (**A**) Western blot showing SREBP-2 protein expression levels in normal chondrocytes treated with 10 ng/ml TGF-β, 10 ng/ml TGF-β plus 1 µΜ SB-431542, and 10 ng/ml TGF-β plus 10 µΜ SB-431542 for 6 h. GAPDH was used as loading control. (**B**) MMP-13 and COL2A1 mRNA expression levels in normal chondrocytes treated with 10 ng/ml TGF-β, 10 ng/ml TGF-β plus 1 µΜ SB-431542, and 10 ng/ml TGF-β plus 10 µΜ SB-431542 for 6 h, normalized to GAPDH. *p<0.05, NS: non significant (**C**) ITGAV mRNA expression in normal and osteoarthritic chondrocytes. GAPDH was used for normalization of the Real Time PCR data. All experiments were performed in duplicate. Data are expressed as mean. *p<0.05 (**D**) A representative western blot showing increased protein levels of ITGAV in osteoarthritic chondrocytes compared to normal, where β-actin was used as loading control.

Since TGF-β was found to upregulate of SREBP-2 mRNA and protein expression, normal chondrocytes were treated with 10 ng/ml TGF-β together with a pharmacological inhibitor of TGF-β receptor (SB-431542), in two different concentrations, 1 µΜ and 10 µM for 6 h. Our results showed that SREBP-2 protein expression levels were downregulated in chondrocytes treated with 10 µM SB-431542 compared to untreated chondrocytes, or treated with TGF-β alone or 1 µM SB-431542 (Figure 4A). Furthermore, we showed that there was no significant difference in MMP-13 and ACAN mRNA expression in treated chondrocytes (Figure 4B).

### Integrin Alpha V (ITGAV) is Upregulated in OA Chondrocytes

Since TGF-β is activated by integrin alpha V (ITGAV), we tested its expression in OA and normal chondrocytes and found that ITGAV mRNA and protein expression levels were significantly elevated in osteoarthritic chondrocytes (p<0.05) (Figures 4C and 4D respectively).

### Blocking of Integrins by cyclo-RGDFV (RGD) Peptide in Osteoarthritic Chondrocytes Leads to Restoration of their Normal Phenotype

**Figure 5:**
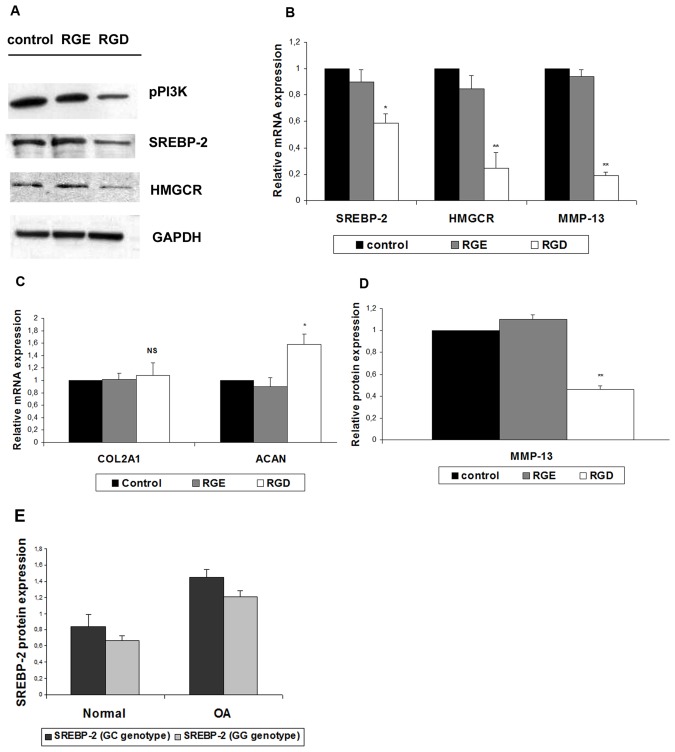
Effect of integrin blockage by cyclo-RGDFV peptide (RGD) on osteoarthritic chondrocytes. (**A**) Effect of treatment of osteoarthritic chondrocytes with 25 µM cycloRGDFV peptide (RGD), control peptide RGE and additional control (untreated cells) for 24 h on SREBP-2, HMGCR and phospho-PI3K protein expression evaluated by Western blot analysis. GAPDH was utilized as loading control. (**B**) SREBP-2, HMGCR and MMP-13, and (**C**) COL2A1 and ACAN mRNA expression in osteoarthritic chondrocytes treated with RGD, RGE and respective control using Real Time PCR. GAPDH was used for normalization of the Real Time PCR data. All experiments were performed in duplicate and data are expressed as mean of two independent experiments. (**D**) MMP-13 expression levels in osteoarthritic chondrocytes following treatment with RGD, RGE and respective control detected by ELISA. *p<0.05, **p<0.001, NS: non-significant (**E**) SREBP-2 protein expression in normal and OA chondrocytes compared to genotypes (GG and GC) of the 1784G/C SNP.

Since PI3K is activated by integrins, and in order to get a more detailed picture of the molecular mechanism implicated in OA pathogenesis, we blocked all integrins upstream of PI3K using the cyclo-RGDFV (RGD) peptide and evaluated the effect on osteoarthritic chondrocytes. Since RGD peptide is a non-specific integrin binding peptide, we used RGE peptide as negative control to show specificity in addition to a no-peptide control. To that regard, osteoarthritic chondrocytes were treated with 25 µM cyclo-RGDFV (RGD) peptide and RGE peptide for 24 hours and the expression levels of SREBP-2, HMGCR, MMP-13, COL2A1 and ACAN were evaluated. Ninety-eight percent (98%) of osteoarthritic chondrocytes treated with the RGD or RGE were viable, as evidenced by the MTT assay confirming thus the good preservation of cell viability (data not shown). We found that SREBP-2, HMGCR and MMP-13 as well as phospho-PI3K mRNA and protein expression levels were significantly reduced in the peptide-treated cells (p<0.001) (Figure 5A, 5B and 5D). Interestingly, ACAN, but not COL2A1 mRNA expression exhibited significantly elevated levels in the treated cells (Figure 5C) (p<0.001) indicating an at least partial reversal of the osteoarthritic phenotype by integrin blockage.

### SREBP-2 1784 G/C SNP Heterozygosity is Associated with Increased SREBP-2 Protein Levels

SREBP-2 genotypes were evaluated in OA and normal cartilage samples. We found that 8/12 OA samples had the heterozygous (GC) genotype, while 4/12 were homozygous for the wild-type allele (GG). Regarding normal samples, 3/6 were heterozygous (GC genotype) and 3/6 were wild-type homozygous (GG). Furthermore, endogenous levels of SREBP-2 protein were evaluated in the above OA and normal samples and we found that GC genotype induced SREBP-2 (Figure 5E) and HMGCR protein expression more efficiently than GG genotype (p<0.05 and ptrend<0.1 respectively). We also found that GC genotype had no effect on the basal expression levels of TGF-β, ITGAV, phospho-PI3K, phospho-Akt, and MMP-13.

### SREBP-2 Interacts with Smad3 in Osteoarthritic Chondrocytes while Smad3 Inhibition Leads to Reduced ACAN Expression

**Figure 6:**
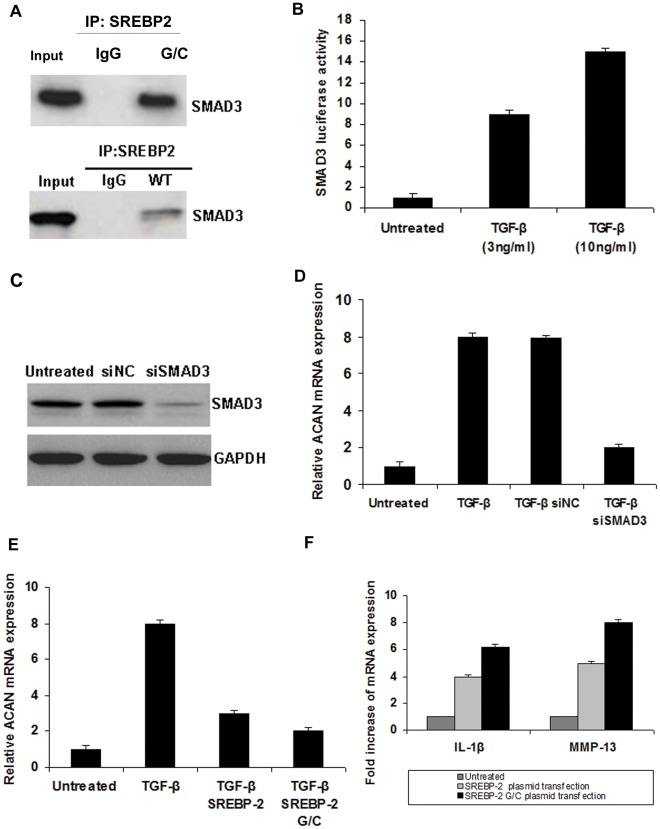
Transfection of chondrocytes with plasmids and interactions between SREBP-2 and Smad3. (**A**) Primary osteoarthritic chondrocytes were transfected by electroporation with SREBP-2 WT and SREBP-2 G/C plasmids. 24 h post transfection we performed immunoprecipitation for SREBP-2 and tested the interaction with Smad3. (**B**) Normal chondrocytes were treated with TGF-β (3, 10 ng/ml) and Smad3 activity was assessed by luciferase activity (GACA) 12-luc plasmid, 12 h post transfection. (**C**) Western blot analysis showing Smad3 protein expression levels in normal chondrocytes treated for 24 h with siRNA control (100 nM) or siRNA against Smad3 (100 nM). GAPDH was used as loading control. (**D**) Normal chondrocytes were treated with TGF-β (10 ng/ml) in the presence of siRNA control (100 nM) or siRNA against Smad3 (100 nM). (**E**) Normal chondrocytes were treated with TGF-β (10 ng/ml) and transfected with SREBP-2 WT or SREBP-2 G/C plasmids and ACAN mRNA expression was tested by real-time PCR. (**F**) SREBP-2 G/C overexpression in normal chondrocytes up-regulated MMP-13 and IL-1β in higher levels in comparison to SREBP-2 WT over-expression (48 h post transfection).

Taking into account the fact that one of the changes observed in OA is ACAN reduction, we focused on molecules that regulate ACAN such as Smad3. Primary osteoarthritic chondrocytes obtained from individuals with GG genotype were transfected with a plasmid overexpressing SREBP-2. Twenty four hours post transfection immunoprecipitation was performed for SREBP-2 and the interaction with Smad3 was assessed. We found that SREBP-2 indeed binds to Smad3 in osteoarthritic chondrocytes (Figure 6A). Moreover, normal chondrocytes were treated with TGF-β and Smad3 activity was assessed by luciferase activity (GACA) 12-luc plasmid showing that TGF-β induces Smad3 activation (Figure 6B). Interestingly, normal chondrocytes treated with TGF-β in the presence of control siRNA or siRNA against Smad3 showed a dramatic reduction of ACAN (Figure 6D). More specifically, Smad3 knockdown (Figure 6C) inhibited TGF-β to up-regulate ACAN mRNA expression (Figure 6D), suggesting that TGF-β activates ACAN only through Smad3.

### SREBP-2 G/C Exhibits Stronger Association with Smad3 and its Overexpression Leads to Dramatic Reduction of ACAN mRNA Expression in Normal Chondrocytes

Since our initial data showed a strong association between *1784G>C* SNP in SREBP-2 gene and OA, we generated a plasmid carrying this specific polymorphism as described previously [31] namely SREBP-2 G/C. To test the functional role of this polymorphism, normal chondrocytes obtained from individuals with GG genotype were transfected with SREBP-2 WT and SREBP-2 G/C plasmids. We observed that overexpression of SREBP-2 G/C in normal chondrocytes resulted in significant up-regulation of constitutive MMP-13 and IL-1β expression in comparison to SREBP-2 WT (Figure 6F). Interestingly, immunoprecipitation performed 24 h post transfection showed that the interaction between SREBP-2 and Smad3 was stronger between SREBP-2 G/C and Smad3 than between SREBP-2 WT and Smad3 (Figure 6A). Finally, simultaneous treatment of normal chondrocytes with TGF-β and transfection with SREBP-2 or SREBP-2 G/C resulted in significant ACAN mRNA reduction, which was more efficient in the SREBP-2 G/C-treated cells than in the SREBP-2 WT-treated cells (Figure 6E) (p<0.05).

## Discussion

There is accumulating evidence linking OA pathogenesis and lipid metabolism prompting many scientists to characterize OA as a metabolic disease [6], [32], [33], [34]. SREBPs are transcription factors regulating lipid metabolism-related genes and although well studied in general, to date there is no knowledge on their involvement in OA.

In order to evaluate the role of SREBP-2 gene in osteoarthritis’ pathogenesis, we performed a genetic association study using a cohort of 1410 OA patients and healthy subjects. We observed that SREBP-2 1784G>C polymorphism was significantly associated with BMI and OA development. Muller et al [21] had previously reported that the above variant, a glycine-to-alanine (G595A) substitution located in COOH-terminal regulating region, could affect the formation and stability of SREBP-2/SCAP complex. More specifically, 595A isoform was found to decrease the stability of the complex compared to 595G isoform affecting the role of SCAP as a sensor in case of cell’s sterol depletion or excess. Taking into consideration the fact that osteoarthritic chondrocytes have been shown to accumulate lipids [34], our finding provides further evidence on the contribution of this polymorphism in the deregulation of lipid transport that characterizes osteoarthritic chondrocytes.

Interestingly, this polymorphism has been previously correlated with atherosclerosis and since atherosclerosis and OA have been thought to share some common characteristics in relation to the implication of lipid metabolism-related genes [32], the latter finding further fortifies this notion.

To further substantiate our finding and conclude on the functional significance of SREBP-2 1784G>C SNP, we transfected normal chondrocytes, obtained from individuals with GG genotype, with SREBP-2 G/C plasmid and found that SREBP-2 G/C over-expression up-regulated IL-1β and MMP-13 expression at higher levels in comparison to SREBP-2 WT over-expression, providing evidence on the involvement of SREBP-2 gene in OA susceptibility in Greek population.

The observed association between SNP 1784G>C and OA occurrence prompted us to investigate whether SREBP-2 expression is altered in OA cartilage compared to normal. Interestingly, our results showed that SREBP-2 mRNA and protein expression levels were significantly elevated in osteoarthritic chondrocytes compared to normal. In addition, HMGCR expression, a SREBP-2 target gene catalyzing a significant step in cholesterol synthesis pathway, was also significantly increased in osteoarthritic compared to normal chondrocytes. Since we found that SREBP-2 was associated with BMI and as obesity is characterized by upregulated cholesterol synthesis [35] we evaluated the effect of sterols on SREBP-2 expression and chondrocytes markers such as MMP-13 and COL2A1. We found that 25-hydroxycholesterol upregulated SREBP-2 protein expression and MMP-13 mRNA expression in OA chondrocytes. All above, provide further evidence on the deregulation of lipid homeostasis in OA.

As PI3K/Akt pathway is an important player in the regulation of lipid metabolism, and previous evidence suggests that SREBP-2 is activated by PI3K/Akt in diabetes, cancer and viral infections [24], we proceeded to investigate whether PI3K/Akt pathway activates SREBP-2 in osteoarthritic chondrocytes. We found, that phospho-PI3K and phospho-Akt protein levels were significantly upregulated in OA chondrocytes compared to normal.

PI3K is an extensively studied protein playing central role in multiple pathological and physiological conditions. PI3K/AKT pathway is known to be activated by growth factors such as TGF-β, as well as integrins [24]. In the present study, we tested both ways of activation and found that both TGF-β and ITGAV exhibited elevated expression levels in osteoarthritic chondrocytes compared to normal. Intrigued by this finding we wanted to further investigate the effect of TGF-β on normal chondrocytes and test whether TGF-β can induce SREBP-2 activation by itself. Our results showed that treatment of normal chondrocytes with TGF-β resulted in SREBP-2, HMGCR, phospho-PI3K and MMP-13 upregulation promoting thus the osteoarthritic phenotype, The use of the pharmacological inhibitor of TGF-β receptor (SB-431542), resulted in downregulation of SREBP-2 expression but had no significant effect on MMP-13 or ACAN expression suggesting the involvement of additional TGF-β-induced pathways. 

Since PI3K is activated by integrins [25] and in order to get a more detailed picture of the molecular mechanism implicated in OA pathogenesis, we decided to block all integrins upstream of PI3K using the cyclo-RGDFV peptide, which blocks several members of the integrin family of proteins, including substrates for laminin and vitronectin (ITG AVβ3) and evaluate the effect on osteoarthritic chondrocytes. Integrin blockage with RGD peptide and no RGE resulted in dramatic reduction of SREBP-2, HMGCR, phospho-PI3K and MMP-13 expression levels accompanied by elevated levels of ACAN, indicating an at least partial reversal of the osteoarthritic phenotype. These results further verify the central role played by integrins in OA pathogenesis and their potential to be used as therapeutic targets.

In an attempt to shed more light upon the molecular mechanism triggered by SREBP-2 activation and taking into consideration the fact that one of the changes observed in OA is ACAN reduction, we focused on molecules that regulate ACAN. ACAN is a direct target gene of Smad3 which is activated by TGF-β [29] and has been shown to interact with SREBP-2 [30]. Our hypothesis was that this interaction should be valid in OA cartilage since our data have so far shown that the alterations seen in OA chondrocytes were TGF-β driven. We confirmed previous data in monkey kidney fibroblast cell lines that Smad3 interacts with SREBP-2 [30] and demonstrated that Smad3 also forms complex with SREBP-2 in OA chondrocytes and that the interaction is stronger in the presence of SREBP-2 G/C genotype. Furthermore, inhibition of Smad3 in normal chondrocytes blocked ACAN upregulation by TGF-β more efficiently when they were transfected with SREBP-2 G/C than with SREBP-2 WT suggesting that TGF-β induces ACAN expression only through Smad3 activation. This observation is in accordance with van der Kraan et al. who showed that Smad3 deficient mice displayed phenotypes similar to human OA [36].

**Figure 7:**
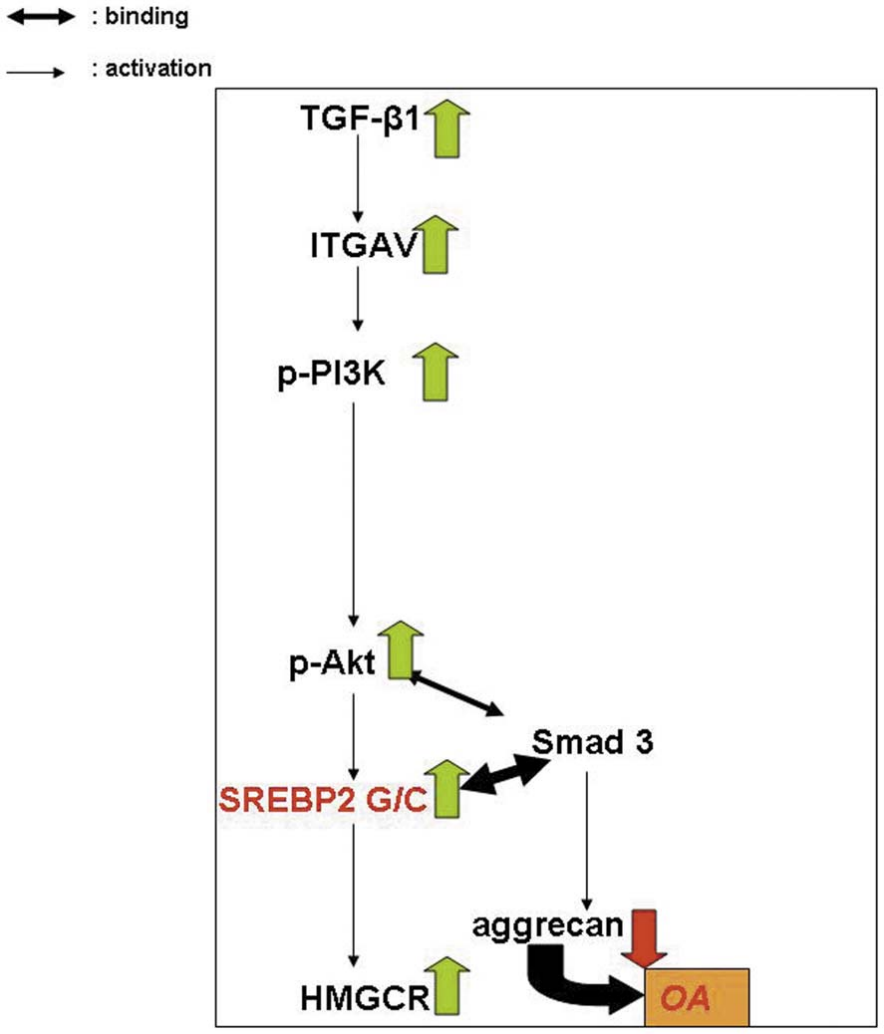
Schematic representation of the molecular pathway activated in osteoarthritic chondrocytes where the involvement of 1784G>C polymorphism is evident. Briefly, in OA, ITGAV and TGF-β upregulation leads to phospho-PI3K and phospho-Akt activation which in turn cause overexpression of SREBP-2 and its target gene HMGCR accompanied by increased levels of MMP-13. Interestingly, chondrocytes expressing the SREBP-2 G/C genotype bind stronger to Smad3 leading to ACAN downregulation, contributing thus to the osteoarthritic phenotype. Furthermore, inhibition of integrins in OA, leads to SREBP-2, HMGCR and MMP-13 downregulation with subsequent elevation of ACAN levels suggesting integrin blockage as a potential molecular target for OA treatment.

In conclusion, we demonstrated, for the first time to our knowledge, the involvement of SREBP-2, a lipid metabolism gene, in OA pathogenesis and provided novel evidence for its TGF-β-induced activation through ITGAV/PI3K/Akt pathway, schematically represented in Figure 7, pointing towards the use of integrin inhibitors as possible molecular targets for osteoarthritis treatment.

## Materials and Methods

### Single Nucleotide Polymorphism (SNP) in SREBP-2 gene Examined in OA and Normal Subjects

#### Study groups

The study included 709 patients with knee OA undergoing knee replacement surgery; 563 women with mean age 66.8±6.2; range 40–92 years and 146 men with mean age 67.4±6.3; range 42–82 years. Height and weight were measured and BMI was computed. Mean BMI of OA patients was 28.96±4.12, range 21.76–43.22. Radiographs were obtained before surgery and graded using the Kellgren - Lawrence system according to the following criteria: grade 1 (doubtful narrowing of joint space and possible osteophytes), grade 2 (definite osteophytes and possible narrowing of joint space), grade 3 (moderate multiple osteophytes, definite narrowing of joint space and some sclerosis and possible deformity of bone ends), grade 4 (large osteophytes, marked narrowing of joint space, severe sclerosis and definite deformity of bone ends) [37]. All patients had a Kellgren Lawrence score ≥2 with over 90% having a K/L score 3 or 4 and were randomly selected. The assessment of the radiographs by two independent expert observers was blinded and the kappa value for inter-reader variation was 0.81 (0.72–0.89). Patients with rheumatoid arthritis and other autoimmune diseases as well as chondrodysplasias, infection-induced OA and post-traumatic OA were not included in the study. The control population consisted of 701 healthy subjects; 384 women with mean age 67.4±8.4; range 48–87 years and 317 men with mean age 64.5±7.2; range 46–88 years. Mean BMI for the control group was 24.8±5.4, range 19.54–27.65. All individuals had no signs or symptoms of arthritis or joint disease (pain, swelling, tenderness or restriction of movement). Because of ethical and financial constraints the knee joints of the controls were not subjected to radiographic analysis. All individuals were of Greek origin living in the region of Thessaly of Central Greece. The study population was in Hardy-Weinberg equilibrium and ethnically homogeneous, which makes the possibility of confounding ethnic heterogeneity less likely. Verbal informed consent was obtained from all participants in the study. The method of obtaining verbal consent was approved by the Institutional Review Board of the University Hospital of Larissa. The study protocol conformed to the ethical guidelines of the 1975 Declaration of Helsinki as reflected in a priori approval by the Local Ethical Committee of the University Hospital of Larissa.

#### SNP selection

We downloaded from the NCBI SNP database (http://www.ncbi.nlm.nih.gov/SNP) all SNPs’ genotype data of SREBP-2 gene based on the genotyped SNPs in European population and found 70 non-synonymous SNPs, out of which only 2 (rs2228314 and rs2228313) had minor allele frequencies (MAF) exceeding 5%. However, as rs2228313 MAF resulted from a low coverage panel of Europeans, we selected to genotype the non-synonymous SNP rs2228314 in SREBP-2 gene located in exon 10 (MAF = 0.36).

#### Genotyping

Genomic DNA was obtained from 3 ml of peripheral blood, using a commercially available kit (Qiagen, Hilden, Germany) according to manufacturer’s instructions. The selected SNP was genotyped using standard polymerase chain reaction and restriction-fragment-length polymorphism methods. The following set of primers was used: (1784G>C): forward 5′-GCCAGTGACCATTAACACCTTTTGA-3′ and reverse: 5′-TCGTCTTCAAAGCCTGCCTCAGTGGCTGGC-3′. Thermal cycling conditions were as follows: 35 cycles of denaturing at 94°C for 1 minute, annealing at 65°C for 1 minute and extending at 72°C for 1 minute. PCR products were digested using MspI enzyme and the restriction fragments were visualized after electrophoresis in 3% agarose gel.

### Osteoarthritic and Normal Articular Cartilage Samples

Articular cartilage samples were obtained from femoral condyles and tibial plateaus of patients with primary OA undergoing knee replacement surgery at the Orthopaedics Department of University Hospital of Larissa. A total of 12 patients were included in this study (10F/2M; mean age 62.5±12.33 years, range 41–76). All osteoarthritic specimens had Mankin score 10–14. Radiographs were obtained before surgery and graded using the Kellgren - Lawrence system according to the following criteria: grade 1 (doubtful narrowing of joint space and possible osteophytes), grade 2 (definite osteophytes and possible narrowing of joint space), grade 3 (moderate multiple osteophytes, definite narrowing of joint space and some sclerosis and possible deformity of bone ends), grade 4 (large osteophytes, marked narrowing of joint space, severe sclerosis and definite deformity of bone ends). All patients had a Kellgren Lawrence score ≥2. The assessment of the radiographs by two independent expert observers was blinded. Patients with rheumatoid arthritis and other autoimmune diseases as well as chondrodysplasias, infection-induced OA and post-traumatic OA were not included in the study. Normal cartilage was obtained from 6 individuals (3F/3M; mean age 42.6±7.6 years, range 32–74) with 0 Mankin score, undergoing fracture repair surgery with no history of joint disease and who did not show clinical manifestations compatible with OA when specifically explored by radiographs. Both patients and healthy individuals’ cartilage samples were obtained upon individuals’ verbal informed consent. The method of obtaining verbal consent was approved by the Institutional Review Board of the University Hospital of Larissa. The study protocol conformed to the ethical guidelines of the 1975 Declaration of Helsinki as reflected in a priori approval by the Local Ethical Committee of the University Hospital of Larissa.

### Primary Cultures of Normal and Osteoarthritic Articular Chondrocytes

Articular cartilage was dissected and subjected to digestion with 1 mg/ml pronase (Roche Applied Science, Mannheim, Germany) for 30 minutes and then the sample was centrifuged and the pellet was subjected to digestion with 1 mg/ml collagenase P (Roche Applied Science, Mannheim, Germany) for 3 h at 37^ο^C. Chondrocytes were counted and checked for viability using trypan blue staining. More than 95% of the cells were viable after isolation. Chondrocytes were cultured with Dulbecco’s Modified Eagles Medium/Ham’s F-12 (DMEM/F-12) (GIBCO, BRL, UK) plus 5% fetal bovine serum (FBS, GIBCO, BRL, UK) and 100 U/ml penicillin-streptomycin, and were incubated at 37^ο^C under a humidified 5% CO_2_ atmosphere until reaching confluence. Chondrocytes were kept in culture for 2 passages, while type II collagen and type I collagen ratio was screened in all samples to exclude dedifferentiation events.

### Chondrocytes’ Transfection with siRNAs or Plasmids

Prior to transfection, we evaluated SREBP-2 genotype in OA and normal samples and compared them with SREBP-2′s protein expression. Primary chondrocytes were treated for 24 h with 100 nM siRNA negative control (Ambion Inc) or 100 nM siRNA against Smad3 (Ambion Inc, USA) and then with TGF-β (10 ng/ml) (Sigma-Aldrich, Missouri, USA) for 12 h. SREBP-2 and SREBP-2 G/C plasmids were constructed according to a previously described method [38]. Transfections with these plasmids in primary chondrocytes were performed using the Amaxa Nucleofector Kit (Lonza, Italy). Smad3 activity was assessed by luciferase activity (GACA) 12-luc plasmid provided by Dimitris Kardassis (School of Medicine, University of Crete).

### SREBP-2–Smad3 Immunoprecipitation Assay

Sub-confluent cells of normal chondrocytes cultures transfected with SREBP-2 WT and SREBP-2 G/C plasmids were lysed in 20 mM Tris-HCl buffer (pH 8) containing protease inhibitors. In separate aliquots of cell lysates was added antibody against SREBP-2. Protein A/G agarose beeds (Thermo Scientific, Rockford, USA) were also added in every sample and they were incubated overnight at 4°C. Agarose beeds were collected and washed with lysis buffer (pH 8). Based-bound proteins were resolved on 4–10% SDS-polyacrylamide gels, and the gels were blotted on nitrocellulose membranes. The membranes were incubated with 5% blocker and then with anti-Smad3 antibody. The membranes were then exposed to photographic film. IgG from the same species as the antibody being used for the IP was used as a negative control.

### RNA Extraction and Quantification of mRNA Expression

Total cellular RNA was extracted from cultured chondrocytes using Trizol reagent (Invitrogen, Life Technologies, Paisley, UK). RNA was further purified using an RNeasy mini kit (Qiagen, Hilden, Germany). Preservation of 28S and 18S ribosomal RNA (rRNA) species was used to assess RNA integrity. All the samples included in the study were with prominent 28S and 18S rRNA components. The yield was quantified spectrophotometrically. Transcription of 1 µg RNA to complementary DNA (cDNA) was performed using the AMVKit (Roche Applied Science, Mannheim, Germany). Quantification of SREBP-2, ITGAV, HMGCR, MMP-13, COL2A1, COL1A1 and ACAN mRNA expression was performed by real-time PCR (ABI 7300, Applied Biosystems Foster, CA). Glyceraldehyde 3-phosphate dehydrogenase (GAPDH) was used as a housekeeping gene. Reactions were done in triplicate using 2 µl of cDNA per reaction. All primers used are shown in Table 3. To quantify the relative expression of each gene, Ct values were normalized against the endogenous reference (ΔCt = Ct target – Ct GAPDH) and were compared with a calibrator using the ΔΔCt method (ΔΔCt = ΔCt sample – ΔCt calibrator).

**Table 3:** Oligonucleotide primers used in real-time PCR assay.

Gene	Forward primer sequence	Reverse primer sequence
SREBP-2	AAGTCTGGCGTTCTGAGGAA	AGGTCCACCTCATTGTCCAC
ITGAV	TTCTCTCGGGACTCCTGCTA	AGCTCCCACGAGAAGAAACA
HMGCR	GTCATTCCAGCCAAGGTTGT	TCCTGTCCACAGGCAATGTA
MMP-13	TGGCATTGCTGACATCATGA	GCCAGAGGGCCCATCAA
COL2A1	ATGACAATCTGGCTCCCAACACTGC	GACCGGCCCTATGTCCACACCGAAT
ACAN	TGAGGAGGGCTGGAACAAGTACC	GGAGGTGGTAATTGCAGGGAACA
GAPDH	ACCACTGTCCACGCCATCAC	TCCACCACCCTGTTGCTGTA
COL1A1	CCTGGGGTCTTCCTTACCTC	CCATGGGGTCAGATGGTATC

### Protein Extraction and Western Blot Analysis

Normal and osteoarthritic chondrocytes were lysed using lysis buffer containing 30mM Tris (pH 7.5), 150mM NaCl, 10% glycerol, 1% Nonidet P-40, and a cocktail of protease and phosphataese inhibitors. Protein concentration was quantified using the Bradford protein assay (Bio-Rad Protein Assay, BioRad, Hercules, CA) with bovine serum albumin as standard. Cell lysates from normal and OA chondrocytes were electrophoresed and separated on a 4–10% Tris-HCl gel (Bio-Rad Protein Assay, BioRad, Hercules, CA) and transferred to a Hybond-ECL nitrocellulose membrane (Amersham Biosciences, Piscataway, NJ) that was probed with anti-ITGAV, SREBP-2, HMGCR, total PI3K, p-PI3K, total Akt and p-Akt (Santa-Cruz Biotechnology Inc. Europe). Signals were detected using suitable immunoglobulin IgG conjugated with horseradish peroxidase (Invitrogen, Life Technologies, Paisley, UK). Anti-β-actin antibody (Sigma-Aldrich, Missouri, USA) and anti-GAPDH antibody (Cell Signaling Technology, Boston, USA) were used as loading controls as indicated in the figure legends.

### ELISA Assay

Culture supernatants from osteoarthritic and normal chondrocytes were harvested and stored frozen at −80°C. TGF-β and MMP-13 were quantitated in cell supernatants by ELISA using Quantikine human TGF-β and MMP-13 immunoassay kits according to the instruction of the manufacturer (R&D Systems, McKinley Place, NE Mineapolis, USA). Samples were measured in duplicate.

### 25-hydroxycholesterol Treatment in OA Chondrocytes

Cells were seeded on six-well plates at a density of 0.3×10^6^ cells/well. Three days post-seeding normal chondrocytes were treated with 25 µΜ of 25-hydroxycholesterol (Sigma-Aldrich, Missouri, USA) or DMEM/F-12 alone for 12 h. Each experiment was conducted in duplicate and the results from 2 wells were averaged and considered as n = 1. RNA and proteins were extracted while real-time PCR and Western blotting analysis were performed.

### TGF-β Treatment in Normal Chondrocytes

Cells were seeded on six-well plates at a density of 0.3×10^6^ cells/well. Three days post-seeding normal chondrocytes were treated with 10 ng/ml of TGF-β (Sigma-Aldrich, Missouri, USA) or DMEM/F-12 alone for 30 min, 2 h, 6 h and 24 h. Each experiment was conducted in duplicate and the results from 2 wells were averaged and considered as n = 1. RNA and proteins were extracted while real-time PCR and Western blotting analysis were performed.

### TGF-β Treatment in Normal Chondrocytes with Subsequent Use of the Pharmacological Inhibitor of TGF-β Receptor, SB-431542

Cells were seeded on six-well plates at a density of 0.3×10^6^ cells/well. Three days post-seeding normal chondrocytes were treated with 10 ng/ml of TGF-β (Sigma-Aldrich, Missouri, USA), 10 ng/ml of TGF-β plus 1 µΜ of SB-431542, 10 ng/ml of TGF-β plus 10 µΜ of SB-431542 or DMEM/F-12 alone for 6h. Each experiment was conducted in duplicate and the results from 2 wells were averaged and considered as n = 1. RNA and proteins were extracted while real-time PCR and Western blotting analysis were performed.

### OA Chondrocytes’ Viability after Treatment with Synthetic Peptides Cyclo-RGDFV (RGD) and RGE

Cells were plated at 1×10^4^ cells/well in a 96-well plate and allowed to attach overnight. The following day, cells were shifted to serum-free medium and then treated with 25 µΜ of cyclo-RGDFV (RGD) peptide (Calbiochem-Novabiochem, UK) for 24 h. Controls included serum-free, peptide untreated cells. A peptide containing an RGE motif (Sigma-Aldrich, Missouri, USA) served as control in addition to a no-peptide control. Cell viability was estimated using the TACS MTT assay kit (R&D Systems, McKinley Place, NE Mineapolis, USA) according to the manufacturer’s instructions.

### Treatment of OA Chondrocytes with Synthetic Peptides Cyclo-RGDFV (RGD) and RGE

Cells were seeded on six-well plates at a density of 0.3×10^6^ cells/well. Three days post-seeding osteoarthritic chondrocytes were treated with 25 µΜ of cycloRGDFV (RGD) peptide (Calbiochem-Novabiochem, UK), RGE peptide (Sigma-Aldrich, Missouri, USA) or DMEM/F-12 alone for 24 h. Each experiment was conducted in duplicate and the results from 2 wells were averaged and considered as n = 1. RNA and proteins were extracted 24 h after treatment with the peptide and were subject to Real Time PCR and Western blotting analysis.

### Statistical Analysis

Genotype distribution, allele frequencies and their association with other variables, such as K/L score, BMI and sex, were analyzed using the chi-square test. In addition, we included in the logistic regression model, variables known to be associated with OA. These variables were age, sex and BMI. Odds ratio (OR) and 95% confidence interval (CI) for relative risks were calculated using Fisher’s exact test when necessary. Probability (p) values quoted were based on two-sided tests. A two-sided p value less than 0.05 was considered as statistically significant.

The minimum detectable ORs under the log additive model with power ≥80% and significance level of 5% were calculated for each comparison using Quanto version 1.2.4 (http://hydra.usc.edu/gxe).

The G-allele frequency (SREBP-2 1784G>C) was set to 77% (the frequency for all 701 of our controls) and the population risk of OA was set to 5%.

Gene expression data were analyzed using unpaired t-test as well as Analysis of Variance (ANOVA) and the Tukey’s Honestly Significant Difference (HSD) as the post hoc test where applicable. Numerical data were expressed as mean ± Standard Deviation (SD). A two sided p value <0.05 was considered as statistically significant.

All statistical analysis was performed using the SPSS software (version 17.0).
